# Serum sPD1 and sPDL1 as Biomarkers for Evaluating the Immune State of Lung Adenocarcinoma Patients

**DOI:** 10.1155/2022/9101912

**Published:** 2022-11-25

**Authors:** Yumin Wu, Hui Guo, Jiawei Yue, Peng Xu

**Affiliations:** ^1^Department of Laboratory Medicine, The Third Affiliated Hospital of Soochow University, Changzhou 213003, China; ^2^Institute of Functional Nano & Soft Materials (FUNSOM), Jiangsu Key Laboratory for Carbon-Based Functional Materials & Devices, Soochow University, 199 Ren'ai Road, Suzhou, 215123 Jiangsu, China; ^3^Department of Orthopaedics, The Third Affiliated Hospital of Soochow University, Changzhou 213003, China

## Abstract

A large proportion of cancer patients benefit from immune checkpoint therapy, while few studies focused on the relationship between soluble PD1 (sPD1) and soluble PDL1 (sPDL1) in serum and immune status of patients. ILC2 and M2 were confirmed to be related to immunosuppression in tumor patients. To determine whether sPD1 and sPDL1 are correlated with the ratio of ILC2 and M2 is helpful to explore the possibility of using sPD1 and sPDL1 as tumor molecular markers. Our results showed an immune balance toward ILC2 and M2-like monocytes in patients with LUAD compared with healthy controls. Meanwhile, decreased CD4^+^T and CD8^+^T cells, as well as elevated PD1^+^CD8^+^T cells, were found in patients with LUAD. The relative mRNA expression levels of ILC2- and M2-characteristic cytokines were also upregulated accompanied by decreased mRNA expression levels of ILC1- and M1-characteristic cytokines in patients with LUAD compared to healthy controls. Moreover, elevated ILC2 frequencies as well as the amount of IL-13 were positively correlated with the amount of sPD1, however, there was no correlation between them and sPDL1. These results suggested that sPD1 and sPDL1 can serve as diagnostic markers to predict the immune state of cancer patients.

## 1. Introduction

Lung cancer is the second most common cancer in terms of new cancer cases and the leading cause of cancer-related deaths worldwide [1]. Non-small-cell lung cancer (NSCLC) accounts for more than 85% of lung cancer cases, and about 70% of NSCLC patients are lung adenocarcinoma (LUAD) type [1]. Surgical treatment is still the best treatment for early lung adenocarcinoma. However, most patients are already in advanced stages of cancer when they are diagnosed. Although chemotherapy can inhibit tumor progression to a certain extent, the efficacy is often limited due to drug resistance or poor physical condition of patients after a certain period of treatment. The emergence of immune checkpoint inhibitors (ICIs) has fundamentally revolutionized the prospect of LUAD treatment and improved the prognosis of LUAD patients [2].

Immune checkpoints are costimulating proteins on the surface of T cells that transmit inhibitory signals [3]. T cell is an important immune cell in human body, and its surface expresses costimulatory immune checkpoint protein [4]. Cancer cells pass through ICIs, blocking the activation of T cells and their cytotoxic effects on the tumor, resulting in immune escape, particularly inhibitors of PD1 (programmed cell death protein 1) and PDL1 (programmed cell death protein ligand 1) [5] PD1 is a cell-surface immunoglobulin which is expressed on T cells and pro-B cells, causing lymphocyte death, and thereby, suppressing immune responses through binding to its receptor PDL1 [6]. Monoclonal antibodies that binds to the PD1 receptor can be used to block its interaction with the ligand. Most of the previous studies focused on PD1 and PDL1 on the cell surface, while few studies focused on soluble PD1 (sPD1) and soluble PDL1 (sPDL1) free in serum or body fluids. Stinne R Greisen et al. have demonstrated that sPD1 can be used as a biomarker reflecting clinically silent but radiographic progression in patients with rheumatoid arthritis [7]. High levels of soluble PD1/PDL1 and lymphocyte-activation gene 3 (LAG-3) were detected in basal cell carcinoma patients, which may serve as prognostic/predictive biomarkers [8]. Also, which related to immune balance, the researchers found that sPD1 and sPDL1 were elevated in gastric carcinoma and renal cell carcinoma, they can also be considered to be an early marker of deterioration [9, 10].

Innate lymphoid cells (ILCs) are lymphocytes composed of natural killer cells (NK) and ILC1, ILC2, and ILC3 that do not express antigen specific receptors but produce cytokines in response to infection or insults that guide and enhance the immune response to the front line of attack [11]. They have been reported to be involved in tumor progression. ILC1 has been shown to have similar potential as NK cells to effectively control metastatic seeding of tumor cells and limit tumor growth [12]. However, in contrast, the presence of ILC2 in tumors is closely associated with impaired antitumor response and poor prognosis [13, 14]. Conversely, there is growing evidence that ILC2 also promotes antitumor responses [15, 16]. The regulatory role of macrophages in the tumor microenvironment (TME) has been well studied. Circulating monocytes, which can migrate into tissues and differentiate into stable tissue-specific macrophages, are not well studied. Circulating monocytes can migrate to the TME and be induced to become tumor-associated macrophages (TAMs) by tumor-derived chemokines and other factors released by infiltrators [17]. They also can be generally divided into two states: (1) M1-like monocytes, which are defined as HLA-DR^+^CD14^+^CD86^+^ and (2) M2-like monocytes, which are defined as HLA-DR^+^CD14^+^CD63^+^/CD204^+^/CD206^+^ [18]. Studies have illustrated that ILC2 can maintain the activation of M2 by their cytokines interleukin- (IL-) 13 and IL-4, which are key to alternative activation pathways of macrophages [19]; together, this leads to an immunosuppressive state in patients with cancer. However, whether ILC2 and M2 macrophages jointly maintain T cell exhaust and their relationship with sPD1 and sPDL1 in peripheral blood of patients with lung adenocarcinoma, it has not been further explored.

In this study, we analyzed sPD1 and sPDL1 in the circulation of patients with LUAD, as well as the proportion of ILC1, ILC2, M1-like monocytes, M2-like monocytes, and T cells in PBMC, and the mRNA levels of characteristic cytokines of these cells. The purpose is to explore whether sPD1 and sPDL1 are related to the imbalance of ILC1/ILC2 and M1-like/M2-like monocytes in the circulation and to provide a theoretical and experimental basis for sPD1 and sPDL1 as tumor predictors.

## 2. Materials and Methods

### 2.1. Patients

Thirty patients (male/female, 17/13, average age 56.20 ± 3.20 years old) who were newly diagnosed with LUAD in the Third Affiliated Hospital of Soochow University (Changzhou First People's Hospital) from June 2020 to January 2021 were selected. Twenty-four healthy subjects (male/female, 16/8, average age 54.27 ± 2.60 years old) were selected as the healthy controls in this study. The patient was excluded who has connective tissue disease, rheumatism, diabetes, and other chronic inflammatory wasting diseases. There were no significant differences in age, gender, underlying diseases, and cardiac function between the two groups of subjects. The study was approved by the Ethics Committee of Soochow University and was performed in accordance with the revised Declaration of Helsinki, 2021. Informed consent was acquired from all individuals. Please refer to Table 1 for clinical information of the patients.

### 2.2. Collection of Peripheral Blood Mononuclear Cells (PBMCs) and Plasma

Peripheral blood samples were collected from all subjects. The plasma and blood cells were then isolated from 500 g for 5 min, and the lower blood cells were collected for isolation of peripheral blood mononuclear cells (PBMCs). The plasma was collected for the detection of sPD1 and sPDL1 by ELISA. The blood cells were subjected to Ficoll–Paque density gradient centrifugation at 2,000 rpm for 20 mins at room temperature. The extracted peripheral blood mononuclear cells (PBMCs) were washed twice with sterile phosphate buffered saline (PBS) (Solarbio, China).

### 2.3. Flow Cytometry Analysis

Human ILC1 was identified as Lin^−^CD127^+^CD161^+^ cell, and ILC2 as Lin^−^CD127^+^CRTH2^+^ cell described in previous studies. Circulating M1-like monocyte was defined as HLA-DR^+^CD14^+^CD86^+^ cell, and M1-like monocyte as HLA-DR^+^CD14^+^CD206^+^ cell described in previous studies. CD4^+^T cell/CD8^+^T cell were defined as CD3^+^CD4^+^/CD8^+^ cell. The extracted PBMCs were incubated with fluorochrome-conjugated primary antibodies for 30 min at 4°C to stain cell surface molecules. The stained cells were then analyzed by flow cytometry (Canto II; BD Biosciences, USA). The following antibodies were used for flow cytometry [all purchased from BioLegend (San Diego, CA, USA) except: FITC-anti-lineage (Thermo Fisher Scientific, USA)], PE-anti-CD127, PerCP-Cy5.5-anti-CD161, APC-cy7-anti-CRTH2, FITC-anti-CD14, PerCP-anti-HLA-DR, PE-cy7-anti-CD86, PE-anti-CD206, PerCP-anti-CD3, PE-cy7-anti-CD8, FITC-anti-CD4, and APC-anti-CD279. The results were analyzed using FlowJo software version X (BD, USA).

### 2.4. RNA Extraction, Reverse Transcription, and Quantitative Real-Time PCR (qRT-PCR)

Total RNA was extracted from PBMCs, and the concentration of RNA was measured by NanoDrop 2000c (Thermo Fisher Scientific, Nidderau, Germany). Then, qRT-PCR was performed with the SYBR Green Premix Ex Taq kit (Takara, Otsu, Japan) based on the CFX96 Touch Real-Time PCR Detection System (Bio-Rad, USA) following the manufacturer's instructions. *β*-Actin was used as the internal control. The primers of the target genes are listed in Table 2.

### 2.5. Enzyme-Linked Immunosorbent Assay (ELISA)

The concentrations of sPD1 and sPDL1 in the plasma of the patients and healthy controls were investigated by ELISA via an immunoassay kit ELISA (R&D Systems, Minneapolis, MN, USA). The mean absorbance of the plate was read by an ELX-800 plate reader (BioTek Instruments, Inc., USA) and then analyzed by Gen5_ (BioTek Instruments, Inc.). The concentrations of sPDL1 and sPD1 were calculated via interpolation from a standard curve.

### 2.6. Statistical Analysis

All results were performed using Prism software version 9.0 (GraphPad Software Inc., USA). The results are expressed as the mean ± standard deviation. Significance was assessed with unpaired Student's *t*-test (RT-PCR, FACS quantification, ELISA) lie in an average value of normal distribution; when the two samples did not meet the normal distribution, we used the Mann–Whitney test. Correlations between variables were determined by Spearman's correlation coefficient. The difference was statistically significant when the *P* value was less than 0.05.

## 3. Results

### 3.1. Elevated sPD1 and sPDL1 Were Found in LUAD Patients Compared with Healthy Controls

**Figure 1:**
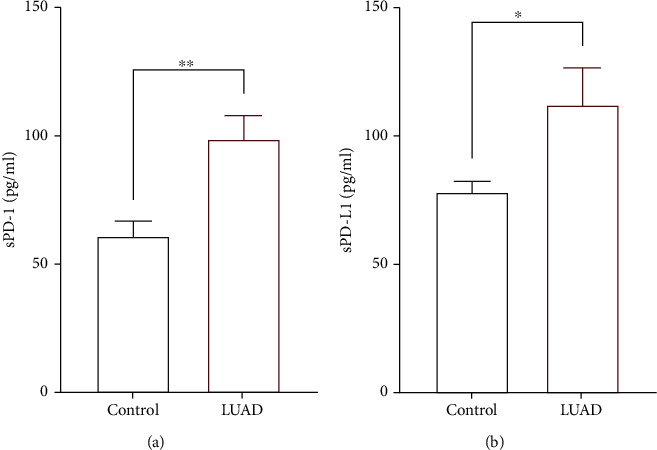
Elevated sPD1 and sPDL1 were found in LUAD patients compared with healthy controls. The protein level of sPD1 (a) and sPDL1 (b) in the serum of patients and controls. Data shown were represented as mean ± SD (all samples were measured in triplicate). ^∗∗^*P* < 0.01, ^∗^*P* < 0.05.

To analyze the levels of sPD1 and sPDL1 in the plasma of patients with LUAD, the protein levels of sPD1 and sPDL1 were detected by ELISA, and we found that sPD1 and sPDL1 levels were elevated in LUAD patients compared with healthy controls (Figures 1(a) and 1(b)). We also analyzed the association of PD1 and PDL1 with overall survival (OS) and postprogression survival (PPS) in patients with LUAD in the TCGA database. The results showed that patients with high expression of PD1 and PDL1 had a worse prognosis, their OS and PPS were shorter than those with low expression (Supplementary Figure 1(a)–1(d)).

### 3.2. The Immune Balance of ILC1/ILC2 Was Biased towards ILC2

**Figure 2:**
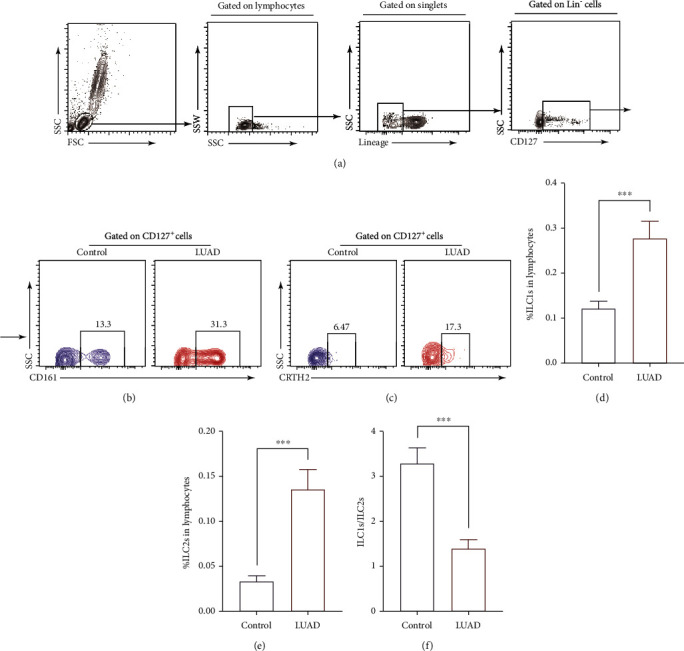
The immune balance of ILC1/ILC2 was biased towards ILC2. (a) Gating strategy of lineage^−^CD127^+^ cells. Gating strategy of ILC1 (b) and ILC2 (c) from patients and healthy controls. Frequency of ILC1 (d) and ILC2 (e) of blood samples from patients and healthy controls. (f) The ratios of ILC1s/ILC2s in blood samples from patients with LUAD and healthy controls. Data shown were represented as mean ± SD (all samples were measured in triplicate). ^∗∗∗^*P* < 0.001.

The percentages of ILC1 and ILC2, defined as lineage^−^CD127^+^CD161^+^ and lineage^−^CD127^+^CRTH2^+^, respectively, were detected by flow cytometry and then calculated as % lymphocytes. The percentages of ILC1 and ILC2 were significantly elevated in LUAD patients compared to healthy controls (Figures 2(a)–2(e)). Next, we compared the immune balance of ILC1/ILC2 in LUAD patients with healthy controls; we found that the balance was biased towards ILC2 in LUAD patients (Figure 2(f)).

### 3.3. The Immune Balance of M1-Like/M2-Like Monocytes Was Biased towards M2-Like Monocytes

**Figure 3:**
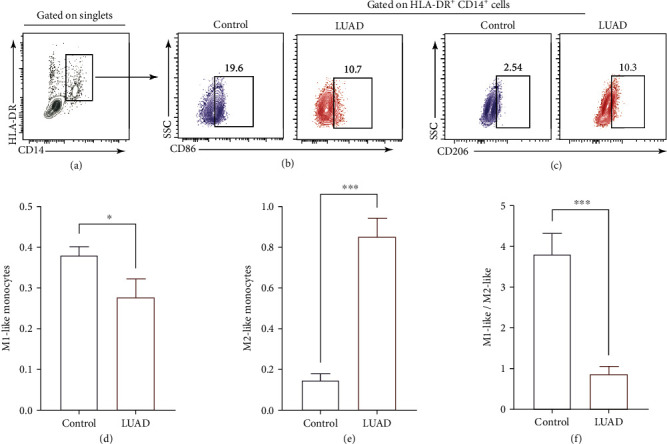
The immune balance of M1-like/M2-like monocytes was biased towards M2-like monocytes. (a) Gating strategy of CD14^+^HLA-DR^+^ cells. Gating strategy of M1-like monocytes (b) and M2-like monocytes (c) from patients and healthy controls. Frequency of M1-like monocytes (d) and M2-like monocytes (e) of blood samples from patients and healthy controls. (f) The ratios of M1-like monocytes/M2-like monocytes in blood samples from patients and healthy controls. Data shown were represented as mean ± SD (all samples were measured in triplicate). ^∗∗∗^*P* < 0.001, ^∗^*P* < 0.05.

Next, we detect the percentages of M1-like/M2-like monocytes, which was defined as HLA-DR^+^CD14^+^CD86^+^ and HLA-DR^+^CD14^+^CD206^+^, respectively, and calculated as %Lymphocytes. The percentages of M1-like monocytes were significantly declined in LUAD patients compared to healthy controls (*P* < 0.05; Figures 3(a), 3(b), and 3(d)). As expert, the percentages of M2-like monocytes were significantly elevated (*P* < 0.001; Figures 3(a), 3(c), and 3(e)). And the immune balance of M1-like/M2-like monocytes was biased towards M2-like monocytes in LUAD patients (*P* < 0.001; Figure 3(f)).

### 3.4. LUAD Patients Showed Immunosuppression and Exhaustion of T Cells Compared with Healthy Controls

**Figure 4:**
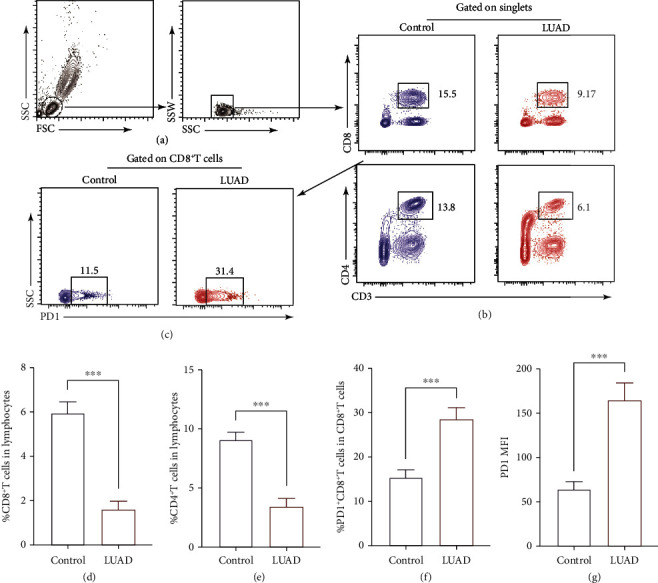
LUAD patients showed immunosuppression and exhaustion of T cells compared with healthy controls. Gating of CD4^+^T/CD8^+^T cells ((a) and (b) and PD1 in CD8^+^T cells (c) from patients and healthy controls. Frequency of CD8^+^T cells (d) and CD4^+^T cells (e) of blood samples from patients and healthy controls. (f) Frequency of PD1^+^CD8^+^T cells of blood samples from patients and healthy controls. (g) The MFI of PD1 in CD8^+^T cells. Data shown were represented as mean ± SD (all samples were measured in triplicate). ^∗∗∗^*P* < 0.001.

As we know, patients with cancer always show an immunosuppressive state; given this, we performed FACS to detect the percentage of CD4^+^T and CD8^+^T cells as well as the exhaustion of CD8^+^T cells. As the results displayed in Figure 4, the percentages of CD4^+^T and CD8^+^T cells in lymphocytes were significantly declined in LUAD patients compared to healthy controls (*P* < 0.001; Figures 4(a), 4(b), 4(d), and 4(e)). Meanwhile, the percentage of PD1^+^CD8^+^T cells in CD8^+^T cells was increased in LUAD patients compared to healthy controls (*P* < 0.001; Figures 4(f) and 4(g)); however, the percentage of PD1^+^CD8^+^T cells in CD3^+^T cells was decreased (*P* < 0.001; Supplementary Figure 2).

### 3.5. Different Expression Levels of ILC-Associated and Monocytes-Associated Cytokines in PBMC of Patients with LUAD

**Figure 5:**
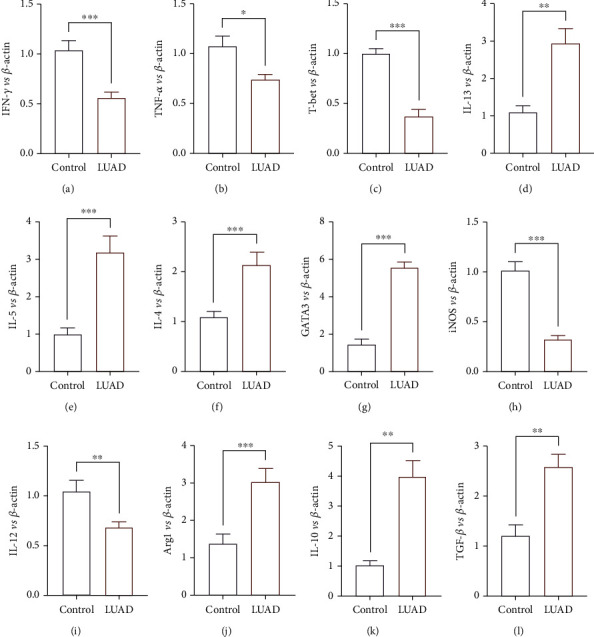
The PBMC levels of ILC-associated and monocytes-associated cytokines in the study population. qRT-PCR analysis of IFN-*γ* (a), TNF-*α* (b), T-bet (c), IL-13 (d), IL-5 (e), IL-4 (f), GATA3 (g), iNOS (h), IL-12 (i), Arg1 (j), IL-10 (k), and TFG-*β* (l) mRNA levels in PBMC from patients and healthy controls. Data shown were represented as mean ± SD (all samples were measured in triplicate). ^∗∗∗^*P* < 0.001, ^∗∗^*P* < 0.01, and ^∗^*P* < 0.05.

The relative expression levels of ILC-associated cytokine genes encoding IFN-*γ*, TNF-*α*, IL-4, IL-13, IL-5, and M1-like/M2-like monocytes-associated cytokine genes encoding iNOS, IL-12, Arg1, IL-10, and TGF-*β*, as well as transcription factors T-bet and GATA3 were determined by the use of real-time PCR. As shown in Figure 5, LUAD patients had elevated expression levels of ILC2- and M2-like monocytes-associated cytokines IL-13, IL-5, IL-4, GATA3, Arg1, IL-10, and TGF-*β* mRNA compared to the healthy controls (Figures 5(d)–5(g), 5(k), and 5(l), *P* < 0.05). IFN-*γ*, TNF-*α*, T-bet, iNOS, and IL-12 (Figures 5(a)–5(c), 5(h), and 5(i), *P* < 0.05) (I) mRNA expressions in the LUAD group were significantly lower than those in the control group (all *P* < 0.05).

### 3.6. The Correlations between the Protein Levels of sPD1and sPDL1 with ILC2 Percentage As Well As the mRNA Level of IL-13

Il-13 is the signature cytokine produced by ILC2. To understand the relationship between IL-13 and ILC2 percentage with the amount of sPD1 and sPDL1 in patients with LUAD, we analyzed the correlations between them. The data indicated that there were positive correlations between sPD1 and ILC2 percentage (Figure 5(a)), as well as the mRNA level of IL-13 (Figure 5(c), in cancer patients, respectively. No correlation was found between sPDL1 with ILC2 percentage (Figure 5(b), or the mRNA level of IL-13 (Figure 5(d)).

## 4. Discussion

Searching reliable biomarkers to predict the efficacy of immune checkpoint inhibitors is the main way to optimize treatment. Studies have examined the expression of sPD1 or sPDL1 to determine patients' sensitivity to ICB therapy [20]. Identifying nonresponders and the patient's immune state will prevent patients from losing access to more effective treatment, but also limits the financial burden of unnecessary treatment. In our present study, we first determined the protein levels of sPD1 and sPDL1 in the serum of patients with LUAD as well as healthy controls. Our results are consistent with those of previous studies [21]; compared with healthy controls, the protein levels of sPD1 and sPDL1 are significantly increased. To explore the correlation between PD1 and PDL1 and prognosis, we then analyzed the association of PD1 and PDL1 with overall survival (OS) and postprogression survival (PPS) based on TCGA database. The results suggested that high expression of PD1 and PDL1 was associated with worse prognosis. These results provide a basis for us to further explore the relationship between sPD1 and sPDL1 and immune status.

As we all know, the immune state of tumor patients presents a state of immune imbalance [22]. ILC, as an important part of the innate immune system, has been the focus of researchers' attention. However, most studies tend to focus on the role of intrinsic lymphatics in tumor tissue in tumor progression [23]. Though studies have proven that PD1 blockade can alleviate the intrinsic PD1 inhibition of ILC2 cells to augment antitumor immunity [24], no study has explored the relationship between the levels of ILCs and the balance of ILC1/ILC2 with sPD1 and sPDL1 in the peripheral blood circulation of tumor patients. Our study performed FACS to detect the percentage of ILC1/ILC2 in the peripheral blood circulation of LUAD patients; we found that compared with controls, the percentage of ILC1 and ILC2 were elevated. However, the balance of ILC1/ILC2 was towards ILC2. This shows us an immune state in which type II immune responses dominate, and cancer has been identified as a disease in which type II immune responses predominate [25].

It is well known that both antitumor M1-like and protumor M2-like TAMs exist in TME, and the antagonistic effects of these M1/M2 subsets on tumors directly affect current strategies to augument antitumor immune responses [26]. In the process of tumor progression, those monocytes and macrophages in tumor tissues, which are actively recruited from circulating, have the potential to change the tumor microenvironment and accelerate tumor progression [27]. Therefore, the proportion of M1/M2 in peripheral blood can be used as an indicator of the patient's immune status. Cancer is a disease in which M2 macrophages predominate [28]. Studies have also proven that ILC2 can maintain the polarization of M2 [19]. Thus, we also verify the balance of M1-like/M2-like monocytes in the peripheral blood of LUAD patients. As the results shown in Figure 3, the balance of M1-like/M2-like monocytes was towards M2-like monocytes. Thus, we speculated that ILC2 and M2 together maintain an immunosuppressive environment. Given this, we then measured the ratios of CD4^+^T cells and CD8^+^T cells in PBMCs of LUAD patients and healthy controls. Our results showed a declined percentages of CD4^+^T cells and CD8^+^T cells in LUAD patients compared with healthy controls (Figures 4(d) and 4(e)). As we know that CD8^+^T cells, as the main participant in the implementation of antitumor immunity, its expression of PD1 was correlated with exhausted signature [29], which was consistent with our results in the peripheral blood of LUAD patients (Figures 4(f) and 4(g)). At the same time, we found that although the proportion of PD1^+^CD8^+^T cells in CD8^+^T cells increased, their proportion in total CD3^+^T cells decreased (Supplementary Figure 2), suggesting that the number of antitumor CD8^+^T cells in cancer patients not only decreased, but that most of them were of the exhausted type.

Commonly, immune cells can perform their functions by their characteristic cytokines [30]. To better verify the immune balance of LUAD patients, we performed RT-PCR to detect the gene expression levels of their characteristic cytokines and transcription factors. The results were inconsistent with the results of cell proportions. As illustrated in Figure 5, the relative expression levels of ILC1-associated cytokine genes and M1-like monocytes-associated cytokine genes as well as their transcription factors T-bet and GATA3 were declined. Their opposites were increased. Although the percentage of ILC1 was increased, the cytokines were declined; we speculated that the function of ILC1 was impaired in the inhibitory environment. And previous studies have shown that although ILC1 can be enriched in precancerous stages, their antitumor function is impaired [31].

**Figure 6:**
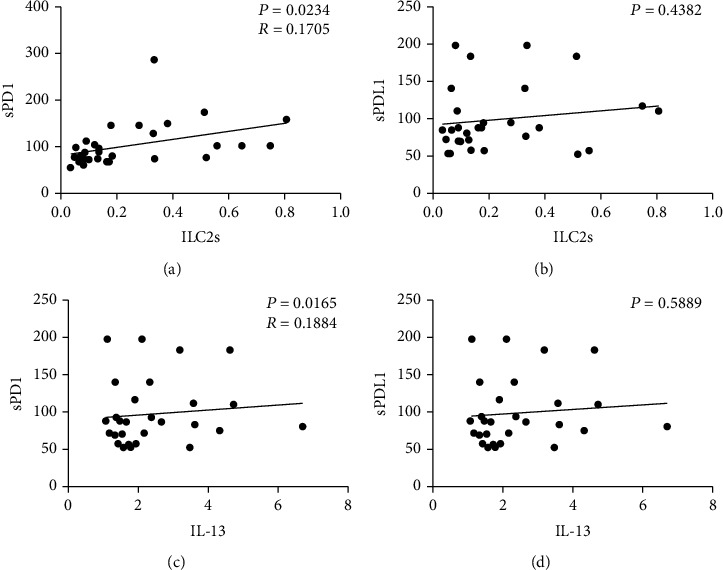
Correlation analysis between sPD1 and sPDL1 with ILC2s and IL-13. The correlation of ILC2s% and sPD1 (a) and sPDL1 (b). The correlation of IL-13 and sPD1 (c) and sPDL1 (d). Data shown were represented as mean ± SD (all samples were measured in triplicate). Ns: not significant.

Studies have shown that ILC2 can promote the polarization of macrophages toward M2 [19], our previous results showed that patients with cancer presented an ILC2 polarized immune state, and our subsequent results also showed the dominance of M2-like monocytes (Figure 4). Therefore, we also detected the mRNA content of M2/M2 characteristic cytokines. Our results are consistent with our flow cytometry results. The mRNA levels of M1-related cytokines in peripheral blood of tumor patients is downregulated, while M2-related cytokines' mRNA levels are upregulated (Figure 5). IL-13 is the most characteristic cytokine of ILC2 [32]. Finally, we compared the proportion of ILC2 and the correlation between the mRNA expression level of IL-13 and the content of sPD1 and sPDL1. The results showed that both ILC2 and IL-13 were related to sPD1 (Figures 6(a) and 6(b)) but had no significant correlation with sPDL1 (Figures 6(c) and 6(d)).

In conclusion, our article verified the correlations between the content of sPD1 and sPDL1 with the immune balance of ILC1/ILC2 and the balance of M1/M2 as well as the exhaustion of T cells in the peripheral blood of LUAD patients, which suggested that sPD1 and sPDL1 can serve as markers to predict the immune state of LUAD patients.

## Figures and Tables

**Table 1:** Clinical information for patients.

Clinical characteristics	Cases	Percentage (%)
Ages		
≥60	21	70%
<60	9	30%
Gender		
Male	17	56.67%
Female	13	43.33%
TNM stage		
I+II	14	46.57%
III+IV	16	53.33%

**Table 2:** The primer sequences for RT-PCR.

Gene	Sequence (5′-3′)	Accession
IFN-*γ*-F	AGTGATGGCTGAACTGTCGC	NM_000619.3
IFN-*γ*-R	ACTCCTTTTTCGCTTCCCTGT	
TNF-*α*-F	GAGGCCAAGCCCTGGTATG	NM_000594.4
TNF-*α*-R	CGGGCCGATTGATCTCAGC	
T-bet-F	TGAGGCTGAGTTTCGAGCAG	NM_013351.2
T-bet-R	CTGGCCTCGGTAGTAGGACA	
IL13-F	CAGAGGATGCTGAGCGGATT	NM_001354993.2
IL13-R	AAACTGGGCCACCTCGATTT	
IL5-F	CAGGGAATAGGCACACTGGA	XM_047417148.1
IL5-R	AGTCTTTCCACAGTACCCCCT	
IL4-F	CTCGCCTACAAAGCCCAGAG	NM_001354990.2
IL4-R	GTGTCCTTCTCATGGTGGCT	
GATA3-F	ACAGAACCGGCCCCTCATTA	XM_047425045.1
GATA3-R	TCCAGAGTGTGGTTGTGGTG	
iNOS-F	CGCATGACCTTGGTGTTTGG	NM_000625.4
iNOS-R	CATAGACCTTGGGCTTGCCA	
IL12-F	GATAAAACCAGCACAGTGGAGGC	NM_000882.4
IL12-R	GCCAGGCAACTCCCATTAGTT	
Arg1-F	GTCTGTGGGAAAAGCAAGCG	NM_000045.4
Arg1-R	CACCAGGCTGATTCTTCCGT	
IL10-F	CTGAGAACCAAGACCCAGACA	NM_001382624.1
IL10-R	GATGTCAAACTCACTCATGGCT	
TGF-*β*-F	CTAATGGTGGAAACCCACAACG	XM_011527242.3
TGF-*β*-R	TATCGCCAGGAATTGTTGCTG	
*β*-Actin-F	TGGCACCCAGCACAATGAA	NM_001101.5
*β*-Actin-R	CTAAGTCATAGTCCGCCTAGAAGCA	

## Data Availability

The data used to support the findings of this study are included within the article.

## Supplementary Materials

Supplementary 1 Supplementary Figure 1. PD1 and PDL1 are poor prognostic factors in LUAD. Lower PD1 expression showed a better OS (a) and PPS (b). Lower PDL1 expression showed a better OS (c) and PPS (d).

Supplementary 2 Supplementary Figure 2. Decreased percentage of PD1^+^CD8^+^T cells in CD3^+^T cells.
