# Supplementary figures and images for: Serum sPD1 and sPDL1 as Biomarkers for Evaluating the Immune State of Lung Adenocarcinoma Patients
Source: J Immunol Res. 2022 Nov 25;2022:9101912. doi: 10.1155/2022/9101912 (PMC9720235; doi:10.1155/2022/9101912)

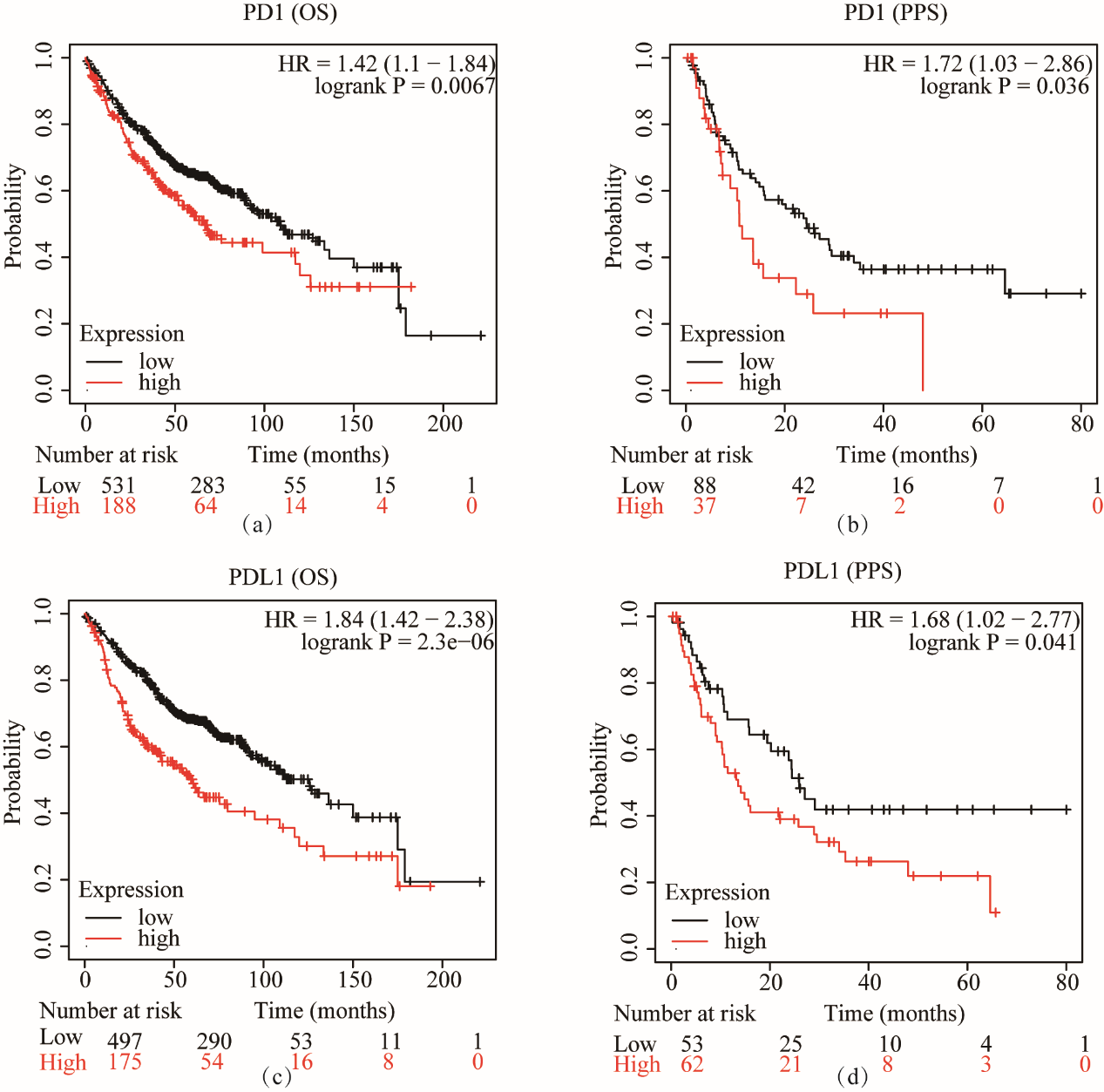

Supplement: Supplementary 1 — Supplementary Figure 1. PD1 and PDL1 are poor prognostic factors in LUAD. Lower PD1 expression showed a better OS (a) and PPS (b). Lower PDL1 expression showed a better OS (c) and PPS (d). [file 9101912.f1.docx]

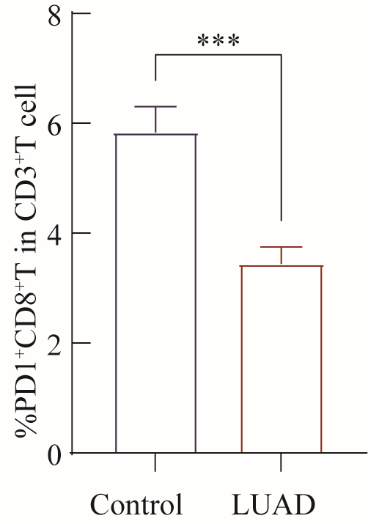

Supplement: Supplementary 2 — Supplementary Figure 2. Decreased percentage of PD1+CD8+T cells in CD3+T cells. [file 9101912.f2.docx]
